# Complete Genome Sequences of Two Outbreak Strains of *Salmonella enterica* subsp. *enterica* Serovar Thompson Associated with Cilantro

**DOI:** 10.1128/genomeA.01365-15

**Published:** 2015-11-19

**Authors:** Craig T. Parker, Steven Huynh, Lisa Gorski, Kerry K. Cooper, William G. Miller

**Affiliations:** a Western Regional Research Center, Produce Safety and Microbiology Research Unit, Agricultural Research Service, U.S. Department of Agriculture, Albany, California, USA; b Department of Biology, California State University-Northridge, Northridge, California, USA

## Abstract

*Salmonella enterica* subsp. *enterica* serovar Thompson strains RM1984 (CADPH-99A2334) and RM1986 (CADPH-99A2345) are associated with a 1999 outbreak in contaminated cilantro. We report here the complete genome sequences and annotation of these two *S*. Thompson strains. These genomes are distinct and provide additional data for our understanding of *S. enterica*.

## GENOME ANNOUNCEMENT

*Salmonella enterica* subsp. *enterica* is a major food-borne pathogen associated with a wide variety of foods and has been classified into multiple groups and specific serovars based on O (lipopolysaccharide) and H (flagellar) antigens (1). *S. enterica* subsp. *enterica* serovar Thompson has been the cause of foodborne outbreaks associated with cilantro, arugula, chicken, beef, bread, and smoked salmon (2–7). *S*. Thompson strains RM1984 and RM1986 were obtained from the California Department of Public Health. These strains were part of a March 1999 outbreak of *S*. Thompson in California associated with the consumption of raw cilantro, which resulted in 76 total cases, including 41 cases from a restaurant and 35 sporadic cases.

Genome sequencing was performed on strains RM1984 (CADPH-99A2334) and RM1986 (CADPH-99A2345) using the Illumina MiSeq to produce 3,530,232 and 4,239,296 reads, respectively. The reads were assembled using the Roche Newbler assembler (version 2.3) to create 68 contigs for RM1984 and 64 contigs for RM1986. The contigs were ordered to the genome of *S*. Thompson strain RM6836 (accession no. CP006717.1) using the Mauve software (8). The contigs and reads were also assembled to the *S*. Thompson reference strain RM6836 genome using the Geneious software (version 8.1). Genome closing utilized a combination of the steps above with the identification of repeated contigs using the Perlscript Contig_extender3 (9). Protein-, rRNA-, and tRNA-coding genes were initially annotated using the NCBI Prokaryotic Genomes Annotation Pipeline (PGAP) (http://www.ncbi.nlm.nih.gov/genomes/static/Pipeline.html). Further annotations were performed in Geneious based on the genome of *S. enterica* subsp. *enterica* serovar Typhimurium LT2 and the O-antigen group C1 locus (accession numbers AE006468.1 and M84642, respectively), with the gene nomenclature for bacterial surface polysaccharides described previously (10).

The complete annotated genome sequences of *S*. Thompson strains RM1984 and RM1986 are 4,708,710 bp and 4,701,355 bp, respectively. The RM1984 genome is predicted to carry 4,380 coding sequences (CDSs), 7 rRNA operons, and 84 tRNAs. The RM1986 genome is predicted to possess 4,376 CDSs, 7 rRNA operons, and 79 tRNAs. Bacteriophages were identified using PHAST (11), and these two strains from the same outbreak were shown to be distinct. Strain RM1984 possesses a Gifsy-1-like prophage, while RM1986 carries a Gifsy-2-like prophage. Each strain possesses four additional remnant prophages. Unlike many other *S. enterica* subsp. *enterica* serovars that carry a virulence plasmid, we did not observe a virulence plasmid in either of these two outbreak strains or in *S*. Thompson strain RM6836 (12). These two genomes are highly syntenic to other *S. enterica* subsp. *enterica* serovars, but their sequences demonstrate variability between strains of *S*. Thompson (12), and, moreover, between two *S*. Thompson strains from the same outbreak.

### Nucleotide sequence accession numbers.

The whole-genome sequences, assemblies, and annotations were deposited with GenBank, BioProject, and BioSample under the accession numbers CP012513, PRJNA292003, and SAMN03959817 for strain RM1984, and CP012514, PRJNA292007, and SAMN03959818 for RM1986.
